# Analysis of the Time and Location of the Silicone Oil Emulsification by Spectral-Domain Optical Coherence Tomography after Silicone Oil Tamponade

**DOI:** 10.1155/2014/372045

**Published:** 2014-05-19

**Authors:** Dominik Odrobina, Iwona Laudańska-Olszewska

**Affiliations:** Klinika Okulistyczna Boni Frates Lodziensis, Ulica Kosynierów Gdyńskich 17, Al. Hippiczna 36 m.2, 94-049 Łódź, Poland

## Abstract

*Purpose*. To estimate localization and the period up to the appearance of small hyperreflective round-shaped droplets using spectral-domain optical coherence tomography (SD-OCT) after pars plana vitrectomy with silicone oil tamponade. *Methods*. A retrospective observational study included 24 patients who had undergone pars plana vitrectomy with silicone oil tamponade for proliferative vitreoretinopathy (PVR) retinal detachment. SD-OCT analysis was performed 1, 3, and 6 months after surgery. We characterized the emulsified silicone oil in the SD-OCT as the small hyperreflective round-shaped droplets. *Results*. In SD-OCT examination, none of the patients had hyperreflective round-shaped droplets visible one month after vitrectomy with silicone oil tamponade. The hyperreflective droplets were found three months after surgery—in one patient above the optic nerve and in five patients intraretinally (in the cystoid spaces). Six months after vitrectomy, the hyperreflective round-shaped droplets were still present in the aforementioned patients' eyes and additionally in 3 eyes above the optic disc. *Conclusions*. Hyperreflective round-shaped droplets were found in a SD-OCT examination 3 months after silicone oil tamponade. The authors suggest that they are most likely the emulsified silicone oil droplets. The authors hypothesize that emulsification and migration of silicone oil begin within 3 months after surgery.

## 1. Introduction

Emulsified silicone oil can penetrate into structures of the eye such as the retina, trabecular meshwork, anterior segment, and optic nerve in patients who were treated with silicone oil tamponade for various vitreoretinal procedures [1–4]. Histopathological examination confirmed that the oil droplets become toxic to the penetrated ocular structures and may result in loss of visual acuity [1, 5, 6].

However, we still know very little about the time when silicone oil becomes emulsificated and its eventual migration to the retina. To date, few reports have shown the presence of emulsified silicone oil droplets intraretinally, subretinally, and beneath the epiretinal membranes in* in vivo* studies, which is optical coherence tomography [7, 8]. However, these studies were only stating its presence while nothing is mentioned about the time in which emulsification and migration of silicone oil occurred.

The purpose of this study is to analyze, in the spectral-domain optical coherence tomography (SD-OCT) examination, the time in which the hyperreflective round-shaped droplets appear after pars plana vitrectomy with silicone oil tamponade. In addition, we also describe the possible localization of the hyperreflective droplets.

## 2. Material and Methods

A retrospective observational study included 24 patients who had undergone pars plana vitrectomy with 1000-centistoke silicone oil (Dorc, Zuidland, The Netherlands) tamponade for proliferative vitreoretinopathy (PVR) retinal detachment. Six patients (6 eyes) additionally underwent 180-degree retinectomy. In 18 patients (18 eyes), internal limiting membrane (ILM) peeling was performed. At the time of surgery, 12 patients were pseudophakic (PCIOL). We performed phacoemulsification with intraocular lens implantation in 12 eyes as a combined procedure with vitrectomy. Four patients have, additionally, diabetes mellitus, nonetheless without evidence of diabetic retinopathy. All retinal surgeries were performed by a single surgeon (D.O.) in local anesthesia.

Patients were excluded if they had undergone a previous vitreoretinal surgery with silicone oil tamponade, silicone oil tamponade of less than 6 months, or different type of silicone oil or had diabetic retinopathy, poor SD-OCT scan quality, or high myopia exceeding −6,0 diopters.

All patients were interviewed and underwent ophthalmologic examinations prior to treatment and 1, 3, and 6 months after vitrectomy with silicone oil tamponade. Examinations included BCVA using standard Snellen eye charts, intraocular pressure, anterior segment, and fundus examination with Volk 78D and 90D lenses (Volk Optical Inc., Mentor, OH, USA). SD-OCT analysis (Spectralis; Heidelberg Engineering, Heidelberg, Germany) was performed 1, 3, and 6 months after surgery. In each patient we performed horizontal line scan through the fovea and 19 B-scans on an area of 4.5×6 mm were done. We compared the results analyzed and performed by 2 ophthalmologists (D.O., I.L.O) and the results were not different.

We characterized the emulsified silicone oil in SD-OCT as the small hyperreflective round-shaped droplets according to the Errera paper [8].

Statistical analyses were carried out using the Pearson product-moment correlation coefficient, Cochran's* Q* test, Fisher's exact test, Wilcoxon signed-rank test, and logistic regression. All the statistical procedures were conducted by means of Stata 12.1 Special Edition (StataCorp LP, College Station, Texas, USA). The significance level was set to be *P* < 0.05.

## 3. Results

The mean age of the 24 patients is 66.92 ± 10.87 years. The mean LogMAR visual acuity before vitrectomy with silicone oil tamponade was 5.12 ± 1.94.

Anatomic success (complete retinal attachment) was noted in all cases. During the last follow-up examination, the retina was still attached in all eyes. The mean LogMAR visual acuity at the final follow-up visit was 2.53 ± 1.13 (Tables 1 and 2). We noted postoperative intraocular pressure (IOP) over 21 mmHg in 7 eyes, all of them receiving topical antiglaucomatous therapy. The IOP was normal in all of these patients at all follow-up visits and all of them still required the same antiglaucomatous drops.

**Figure 1:**
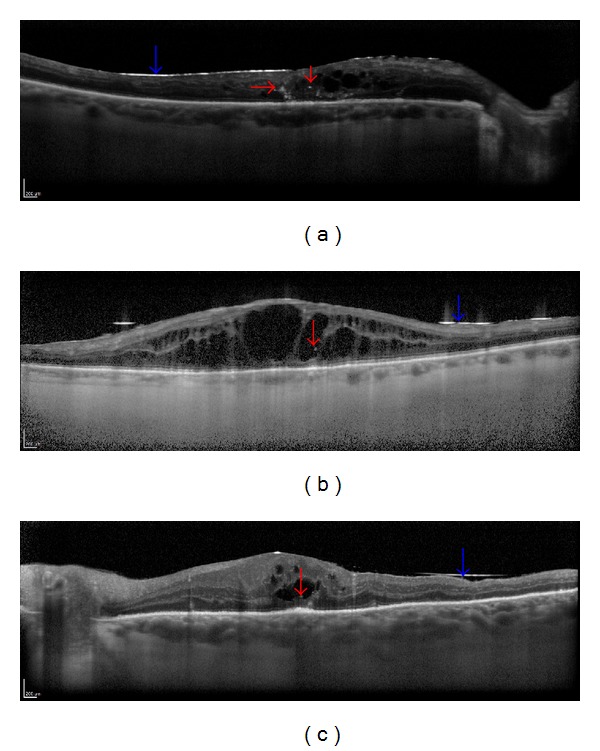
Spectral-domain optical coherence tomography (SD-OCT) showing hyperreflective droplets of emulsified silicone oil visible intraretinally—in the cystoid spaces (red arrows) in 3 cases after vitrectomy with silicone oil tamponade (blue arrow) ((a)–(c)).

**Figure 2:**
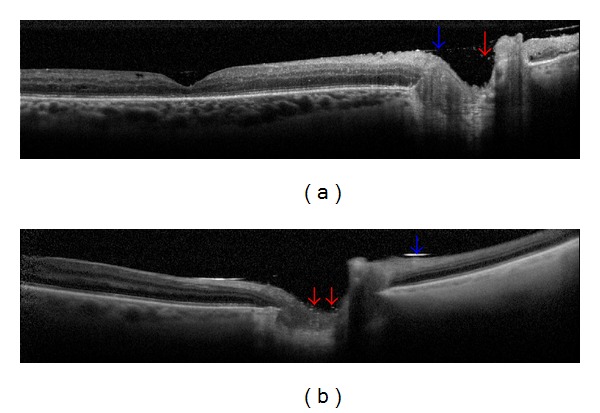
SD-OCT showing hyperreflective round-shaped droplets (red arrow) between the visible hyperreflective line of silicone oil (blue arrow) and the optic nerve in 2 cases ((a)-(b)).

In SD-OCT examination, none of the examined patients had hyperreflective round-shaped droplets visible one month after vitrectomy with silicone oil tamponade. The hyperreflective droplets of emulsified silicone oil were found three months after surgery; in one patient the droplets were detected between the visible hyperreflective line of silicone oil and the optic nerve; in five participants the emulsified silicone oil was found intraretinally (in the cystoid spaces) (Figure 1). Six months after vitrectomy, the hyperreflective round-shaped droplets were still present in the aforementioned patients' eyes and additionally in 3 study participants between the hyperreflective line of silicone oil and the optic disc (Figure 2).  Table 3 records detailed information about each patient with visible hyperreflective round-shaped droplets in SD-OCT examination. Importantly, 11 patients presented cystoid macular edema (CME) at baseline and during the follow-up examinations. In 2 patients epiretinal membrane was found in the follow-up study 3 months after surgery (in these patients ILM peeling was not performed).

The persistence of the hyperreflective, tiny, round-shaped droplets of emulsified silicone oil between its hyperreflective line and the optic nerve was conditioned by the examined patients' age; the discussed droplets of emulsified silicone oil were significantly more frequent in younger participants of the study (*P* = 0.014), prevalence of glaucoma patients (*P* = 0.002), absence of CME (*P* = 0.034), and implementation of ILM peeling procedure (*P* < 0.001). The persistence of the droplets of emulsified silicone oil in the intraretinal area (in the cystoid spaces) was statistically determined only by the occurrence of CME (*P* = 0.020).

## 4. Discussion

In this study we submit that the SD-OCT examination can show the localization and allow detection of the time when small hyperreflective round-shaped droplets, which may be defined as emulsified silicone oil, appear.

Silicone oil has been used as an intraocular tamponade for many years and is considered to be relatively safe. However, its toxicity to the retina or the optic nerve has also been reported [4, 5].

The presence of intraretinal silicone oil emulsification in optical coherence tomography has been described in only a few articles [7, 8]. Errera et al. analyzed a few eyes in SD-OCT in which they demonstrated tiny hyperreflective spherical bodies intraretinally and underneath epiretinal membrane in eyes with silicone oil tamponade. They showed, on the basis of their observation, that these areas are most likely emulsified silicone oil. We are finding similar areas in our study and also assumed that this may be emulsified silicone oil.

The duration in which the emulsification of silicone oil occurred and the mechanism of penetration into the retina are still unclear [7, 9, 10]. Some authors demonstrated the presence of silicone oil intraretinally only in the eyes in which retinal architecture was compromised in patients with retinotomy or the presence of oil subretinally [1, 11]. Errera et al. also demonstrate the presence of emulsified silicone oil droplets intraretinally in eyes in which retinotomy was not performed and in eyes without silicone oil under the retina. In our study, hyperreflective tiny droplets were shown intraretinally in 3 patients after retinectomy, but in 2 patients the retina architecture was intact.

In each of the 5 patients diagnosed with cystoid macular edema, the hyperreflective areas were shown at the edges of cystoid spaces. Errera et al. also noted the presence of tiny hyperreflective spherical bodies in cystoid spaces and on the edges of retinotomy sides, where retina was also with edema. Inflammation and long-lasting retinal detachment can cause retinal edema. In immunochemistry examinations, some authors found intraretinal macrophages containing phagocytosed silicone oil. Perhaps the extension of retinal layers in macular edema may lead to the penetration of macrophages with small droplets of emulsified silicone oil into the external and internal layers of the retina.

According to some authors, intact inner limiting membrane may be a barrier to the penetration of oil into the retina [7, 12]. On the other hand, ILM peeling is regarded as prophylaxis of epiretinal membrane formation [13]. In 18 patients who underwent ILM peeling, only 3 had hyperreflective droplets of emulsified silicone oil inside the retina. In contrast, in 2 patients with epiretinal membranes we did not find hyperreflective droplets in SD-OCT examinations. Analyzing the previous histological examinations, it may be noted that emulsified silicone oil is often present on or beneath the epiretinal membranes [1, 14]. In the SD-OCT study, Errera et al. demonstrated spherical hyperreflective bodies (emulsified silicone oil) underneath epiretinal membranes in 7 patients. They also showed that, in 60% of cases, patients with hyperreflective droplets intraretinally corresponded with the epiretinal membrane. Also, Wickham et al. found that, in all eyes with silicone oil inside the retina, the epiretinal membrane is present. It follows that emulsified silicone oil has a strong affinity to the epiretinal membranes.

Emulsification of silicone oil and migration into the eye structures may occur at different times after vitrectomy with silicone oil tamponade. So far, there are no studies that show the time in which the emulsified silicone oil can penetrate into the human retina. Ohira el al. showed that emulsified silicone oil injected into rabbit's eyes migrated to the inner retinal layers after 1 week [10]. The migration of emulsified silicone oil was also described by the same author in the short time observation from anterior chamber of rabbit [15]. On the other hand, in a histopathologic study, the mean duration of silicone oil tamponade before enucleation was 5 years [1]. Errera et al. only demonstrated emulsified silicone oil in the SD-OCT study, but we do not know since when there was emulsification of oil in the eye [8]. In our study we found hyperreflective tiny droplets visible in high resolution SD-OCT 3 months after silicone oil tamponade. So, we can therefore hypothesize that emulsification and migration of silicone oil occurred after 3 months. Actually, the SD-OCT could give us* in vivo* high resolution cross-sectional images of the retina and also give us more information of the location and time of silicone emulsification.

There are several limitations to this study. Our study has a small sample size. We cannot definitively determine that the hyperreflective round-shaped droplets are emulsified silicone oil. In our opinion, the hypothesis concerning hyperreflective structures as emulsified silicone oil cannot be definitively confirmed without histopathological examinations.

We hypothesize that hyperreflective round-shaped droplets found in SD-OCT correspond with emulsified silicone oil. The authors hypothesize that emulsification and migration of silicone oil begin within 3 months after surgery.

## Figures and Tables

**Table 1:** Descriptive statistics for age, intraocular pressure (mmHg), and logMAR values at baseline and at final examination, taking place after 6 months, in 24 examined patients.

	M*	Me^†^	SD^‡^	SE**	95% CI^††^	CV^‡‡^	Min.–Max.	*P* value***
Patients' age (years)	66.92	64.00	10.87	2.22	62.33–71.51	16.24%	46–90	
Intraocular pressure (mmHg)								
At baseline	13.04	14.00	2.91	0.59	11.81–14.27	22.32%	8–19	
At final exam	15.17	16.00	4.59	0.94	13.23–17.11	30.29%	5–21	
LogMAR								
At baseline	5.12	5.76	1.94	0.40	4.30–5.94	37.84%	2.30–6.91	<0.001
At final exam	2.53	2.65	1.13	0.23	2.06–3.01	44.46%	0.92–4.61	

*M: mean value.

^†^Me: median value.

^‡^SD: standard deviation.

**SE: standard error.

^††^95% CI: 95% confidence interval.

^‡‡^CV: coefficient of variation.

***The Wilcoxon test was performed.

**Table 2:** Descriptive statistics for age, intraocular pressure (mmHg), and logMAR values in eight patients with hyperreflective round-shaped droplets during the follow-up SD-OCT examination.

	M*	Me^†^	SD^‡^	SE**	95% CI^††^	CV^‡‡^	Min.–Max.
Patients' age (years)	64.25	63.50	9.22	3.26	56.54–71.96	14.36%	46–75
Intraocular pressure (mmHg)							
At baseline	13.13	14.00	1.55	0.55	11.83–14.42	11.83%	11–15
At final exam	17.50	18.00	2.88	1.02	15.09–19.91	16.45%	12–21
LogMAR							
At baseline	4.69	4.95	2.38	0.84	2.70–6.68	50.72%	2.30–6.91
At final exam	2.06	1.96	0.94	0.33	1.27–2.84	45.52%	0.92–3.22

*M: mean value.

^†^Me: median value.

^‡^SD: standard deviation.

**SE: standard error.

^††^95% CI: 95% confidence interval.

^‡‡^CV: coefficient of variation.

**Table 3:** Detailed information about each patient with hyperreflective round-shaped droplets found in the SD-OCT examination in follow-up study.

Age (years)	DM	Retinectomy	Glaucoma	CME	ILM peeling	Location of SO 1 month postop.	Location of SO 3 months postop.	Location of SO 6 months postop.
73	−	+	+	+	+	−	Intraret.	Optic nerve, intraret.
71	−	+	+	+	−	−	Intraret.	Intraret.
75	−	−	+	+	+	−	Intraret.	Intraret.
64	−	−	−	−	+	−	—	Optic nerve
46	−	−	−	+	−	−	Intraret.	Intraret.
63	−	+	+	+	+	−	Intraret.	Intraret.
60	−	−	+	+	+	−	—	Optic nerve
62	−	−	+	−	+	−	Optic nerve	Optic nerve

DM: diabetes mellitus; CME: cystoid macular edema; ILM: internal limiting membrane; SO: silicone oil; postop.: postoperatively; intraret.: intraretinally.
